# Preparation and Characterization of Poly(Lactic Acid)/Poly (ethylene glycol)-Poly(propyl glycol)-Poly(ethylene glycol) Blended Nanofiber Membranes for Fog Collection

**DOI:** 10.3390/membranes13010032

**Published:** 2022-12-27

**Authors:** Muhammad Omer Aijaz, Seong Baek Yang, Mohammad Rezaul Karim, Mohd Hafiz Dzarfan Othman, Ibrahim Abdullah Alnaser

**Affiliations:** 1 Advanced Membrane Technology Research (AMTEC), Faculty of Chemical and Energy Engineering (SCEE), Universiti Teknologi Malaysia (UTM), Skudai 81310, Malaysia; maijaz@ksu.edu.sa; 2 Department of Biofibers and Biomaterials Science, Kyungpook National University, Daegu 41566, Republic of Korea; yesyesbaek@gmail.com; 3 Department of Mechanical Engineering, College of Engineering, King Saud University, Riyadh 11421, Saudi Arabia; ianaser@ksu.edu.sa

**Keywords:** poly(lactic acid), electrospinning, fog water harvesting, nanofibrous membrane, hydrophilicity, hydrophobicity, PEG-PPG-PEG

## Abstract

Fog is a resource with great potential to capture fresh water from the atmosphere, regardless of the geographical and hydrological conditions. Micro-sized fog collection requires materials with hydrophilic/phobic patterns. In this study, we prepared hydrophilic poly(lactic acid) (PLA)/poly(ethylene glycol)-poly(propyl glycol)-poly(ethylene glycol) (PEG-PPG-PEG) blended nanofiber membranes with various PEG-PPG-PEG concentrations by electrospinning. Changes in the morphological and chemical properties, surface wettability, and thermal stability of the PLA/PEG-PPG-PEG composite nanofiber membranes were confirmed using field-emission scanning electron microscopy, Fourier-transform infrared spectroscopy, X-ray diffraction, contact angle testing, and thermogravimetric analysis. As the PEG-PPG-PEG content of the nanofiber membranes increased, their hydrophilicity increased. Water stability, membrane porosity, and water transport rate tests were also conducted to observe the behavior of the hydrophilic PLA nanocomposite membranes in aqueous media. Finally, we applied the PLA-based membranes as fog collectors. As the PEG-PPG-PEG content of the nanofiber membranes increased, their ability to collect fog increased by over 40% compared with that collected by a pure PLA membrane. The prepared membranes not only improve the ability of fog collectors to harvest water but also broaden the use of PLA-based membranes in multiple applications, including tissue engineering, drug delivery, scaffolds, and pharmaceuticals.

## 1. Introduction

The solution to the world’s freshwater issues is hidden in nature itself, as the atmosphere holds nearly 10% of all fresh water on Earth in the form of fog. Among the various water resources currently available, fog may be considered the most promising source of fresh water collected from the atmosphere, regardless of the geographical and hydrological conditions. Natural examples reveal that fog can be collected by the micro-sized alternating hydrophilic and hydrophobic patterns of desert beetles, and the hydrophilic knots of spiders and groves of cactus spines can capture, collect, and transport moisture [1]. Considering their merits, electrospun nanofiber membranes are an ideal choice for obtaining these microstructures.

The membrane technology research community has gained much awareness regarding the devastating effects of petroleum-based polymers on the environment and public health. In 2019, European Bioplastics and the Nova-Institute issued general recommendations encouraging the chemical industry to develop sustainable products by increasing the use of bio-based materials [2]. As a result of this awareness, biopolymers have gradually been developed to replace petroleum-based polymers. Biodegradable polymers such as poly(vinyl alcohol) (PVA), poly(vinyl acetate), polycaprolactone, poly(lactic acid) (PLA), chitosan, poly(ethylene glycol) (PEG), and cellulose acetate, all of which are attractive alternatives to petroleum-based polymers, have recently gained increased interest owing to their biocompatibility and ecological advantages [3,4,5]. Among these materials, PLA is considered the biopolymer with the greatest potential to replace petroleum-based polymers because of its abundant availability, good processability, biocompatibility, and low cost. Intrinsically, PLA is a hydrophobic material that exhibits poor toughness, low thermal stability, a slow degradation rate, and low side-chain group reactivity. Commercially, PLA is known as an eco-friendly polymer that is used to produce short-shelf-life products, such as packaging films, food cutlery, and bottles; biomedical products, such as joints, screws, scaffolds, and artificial blood vessels; and agricultural products, such as mulching films [5].

Recent advances in electrospinning methods have led to the production of ultrafine nanofiber membranes with unique features for various applications, such as wound healing, protective clothing, wastewater treatment, energy generation, drug delivery, tissue engineering, and biomedical purposes [6,7,8]. The production of fibers through the electrospinning method is based on the electrostatic drawing of jets of polymer solutions. Initially, droplets of the polymer solution are stretched under a strong applied voltage to produce nano- or microfibers, which are then deposited on the collector [9]. Owing to their extracellular matrix, large specific surface area, high porosity, small pore size, and appropriate mechanical features, PLA-based electrospun nanofibers have gained much interest for their potential use in medical implants, wound dressings [10], tissue engineering [11], scaffolds [12], drug delivery carriers [13], and water treatment applications [14].

**Figure 1:**
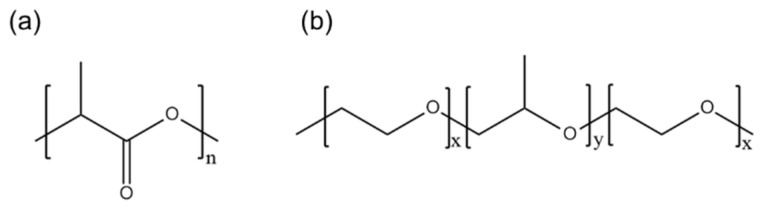
Chemical structures of (**a**) PLA and (**b**) PEG-PPG-PEG.

Good hydrophilicity is a prerequisite for the effective utilization of electrospun nanofiber membranes in the abovementioned applications. A hydrophilic surface allows cells to easily interact with the membrane surface in biomedical applications. Similarly, in water treatment applications, a hydrophilic surface improves pollutant removal by allowing the aqueous medium to wet and penetrate the material. In fog water-collection applications, the hydrophilic part of the membrane captures and collects water, whereas the hydrophobic part facilitates the transport of water into the reservoir. For instance, Uddin et al. [15] utilized the superhydrophobic–hydrophilic characteristics of electrospun polyacrylonitrile (PAN) and poly(methyl methacrylate) nanofibers for atmospheric fog water harvesting. When hydrophilic nanomaterials and a hydrophobic PAN domain were combined, the efficiency of the water-collection mechanism of the resulting material increased because water condensed on the hydrophilic micro- and nanoparticles and quickly rolled off the hydrophobic nanofibers. Similarly, Knapczyk–Korczak et al. [16] introduced hydrophilic polyamide 6 (PA6) electrospun nanofibers to Raschel meshes, a common type of fog collector. In this study, the Raschel meshes were directly coated with the PA6 nanofibers to create a hierarchical structure, leading to enhancements in the surface area of the membrane and its ability to capture water droplets from fog. Other researchers have achieved a threefold increase in the rate of water collection from fog with the addition of hydrophilic nanofibers. Recently, for example, Hou et al. constructed a double-layer electrospun membrane featuring a bottom layer made of hydrophobic PLA and a top layer made of PVA particles with a water-chestnut-like surface morphology [17]. This hydrophilic and hydrophobic membrane successfully collected 57 mg·cm^−2^·h^−1^ water from fog. PVA forms excellent water-chestnut-like structures via electrospinning; however, its fog collection application is limited by its high hydrophilicity because it dissolves immediately upon contact with water. Therefore, PLA, as a hydrophobic polymer (contact angle > 120°), must be modified or functionalized to improve its wettability (contact angle < 90°) (Figure 1a).

Pre- and post-electrospinning methods are mainly applied to develop hydrophilicity in PLA-based electrospun nanofibers. In the pre-electrospinning method, hydrophilicity is introduced during the preparation of the electrospinning solution by blending with hydrophilic polymers or agents. In the post-electrospinning method, surface hydrophilicity is introduced after the fabrication of electrospun membranes by using various post-treatments. In the pre-electrospinning method, hydrophilic polymers, such as PEG, chitosan, and PVA, are mixed with the PLA solution prior to electrospinning; in the post-electrospinning method, different wet chemical functionalization methods, such as hydrolysis, grafting, plasma treatment, coating, or layer-by-layer assembly [18,19,20,21], are applied to the surface of the electrospun PLA membranes. Post-electrospinning methods alter only the fiber surface, which is preferred from the perspective of final scaffold properties [22]. For instance, a study published by Milanesi et al. [23] applied the pre- and post-electrospinning methods to improve the antibacterial activity of PLA membranes for wound dressing applications by employing essential oils, followed by coating with chitosan, to convert the hydrophobic PLA membrane surface into a hydrophilic one. The hydrophilic surface enhanced the antibacterial potential of the essential oils by promoting cell adhesion and proliferation. The authors found that the pre-electrospinning method not only altered the hydrophilicity of the resulting membrane but could also improve its other properties by incorporating various nanoparticles, drugs, and polymers. Moradkhannejhad et al. prepared curcumin-loaded PLA nanofibers using the electrospinning technique to confer drug-release ability for wound dressing applications [24]. The hydrophilicity of the PLA nanofibers was achieved by mixing different concentrations of PEG, and a platform that could accelerate drug release for wound dressing applications was established by loading different amounts of curcumin. Aboutalebi Anaraki et al. prepared PLA/PEG/multiwalled carbon nanotube (MWCNT) nanofibrous scaffolds to obtain an anticancer drug delivery system [25]. In this study, PEG and MWCNT were added at the pre-electrospinning stage; the former tailored the properties of the PLA nanofibers, and the latter controlled the drug-release properties of the resulting scaffolds.

A PLA-based biosensor was developed by modifying PLA nanofibers with PLA-block-PEG (PLA-*b*-PEG) copolymers to enhance its ability to contain biologically active materials on its surface; the surface of the resulting biosensor was then functionalized with biotin. The PLA-*b*-PEG copolymers increased the availability of biotin on the PLA surface by 60%; without PLA-*b*-PEG, only 11% biotin was available on the surface of the biosensor. The water stability of the biotin-loaded biosensor was tested, and PLA modified with PLA-*b*-PEG was found to migrate to the aqueous phase. The authors concluded that the prepared biosensor was applicable as a short-term sensing substrate for sample solutions [26]. In another study, a low-molecular-weight Pluronic based on a poly(ethylene oxide) (PEO) copolymer, was blended with PLA to increase the hydrophilicity of a PLA membrane. The membrane surface was distorted with numerous holes after immersion in water for 24 h because of the leaching of PEO from the fibers and its dissolution in water [27].

The blending of PEG and low-molecular-weight hydrophilic block copolymers into a PLA solution is a successful strategy to increase the hydrophilicity of PLA electrospun membranes. Membranes blended with low-molecular-weight hydrophilic block copolymers show relatively better water stability than those blended with homopolymers. Therefore, a high-molecular-weight tri-block copolymer of PEG may be a feasible option to improve the water stability of hydrophilic PLA membranes under long-term exposure to aqueous media.

PEG-*b*-poly(propylene glycol)-*b*-PEG (PEG-PPG-PEG) is a tri-block copolymer that acts as a nonionic surfactant. The properties of PEG-PPG-PEG depend on the hydrophilic (PEG)/hydrophobic (PPG) ratio (Figure 1b). A higher hydrophilic content (PEG content) results in better oil- and water-stabilization properties, which could help solids disperse and improve the bioavailability of poorly water-soluble compounds, such as drugs, in pharmaceutical and biomedical applications [28].

In this study, we aimed to modify the nanofibrous surface of PLA with a PEG-PPG-PEG tri-block copolymer for the first time using a pre-electrospinning approach. Changes in the morphological and chemical properties, surface wettability, and thermal stability of the PLA/PEG-PPG-PEG composite nanofiber membranes were confirmed using field-emission scanning electron microscopy (FE-SEM), Fourier-transform infrared (FT-IR) spectroscopy, X-ray diffraction (XRD), contact angle (CA) testing, and thermogravimetric analysis (TGA). Moreover, water stability, membrane porosity, and water transport rate (WTR) tests were conducted to observe the behavior of the hydrophilic PLA nanocomposite membranes in aqueous media. Finally, we applied the PLA-based membranes as fog collectors. The introduction of hydrophilicity to the PLA nanofibers improved the water collection efficiency of the resulting membranes, thus confirming that modified PLA is a potential candidate material for atmospheric fog water collection. The combination of the electrospinning technique and nanofiber modification could potentially enhance and expand the uses of PLA-based membranes not only in water harvesting but also in tissue engineering, drug delivery, scaffolds, and pharmaceutical applications.

## 2. Materials and Methods

### 2.1. Materials

PLA (M*_w_* = 163,000, 1.25 g·mL^−1^, LX175^®^) was purchased from Filabot, Co. Ltd. (Barre, VT, USA). PEG-PPG-PEG (M*_n_* = ~14,600, 1.018 g·mL^−1^, Pluronic^®^ F-108), dichloromethane (DCM), and dimethylformamide (DMF) (analytical-reagent grade, ≥99.8% purity) were purchased from Sigma Aldrich (St. Louis, MO, USA).

### 2.2. Preparation of the PLA and PLA/PEG-PPG-PEG Blend Solutions

The PLA and PEG-PPG-PEG blend solutions were prepared into electrospun nanofibrous membranes. Initially, 12% (*w*/*v*) PLA was dissolved in DCM at 50 °C for 1 h to prepare a clear and transparent PLA solution. Then, different concentrations (1, 5, 7, 10, and 30% (*w*/*v*)) of PEG-PPG-PEG were added to DMF at 50 °C for 30 min to prepare the PEG-PPG-PEG solutions. The two solutions were mixed at a ratio of 4:1 and stirred for 12 h to ensure that a homogeneous solution was formed.

### 2.3. Electrospinning of PLA and PLA/PEG-PPG-PEG Blended Nanofiber Membranes

To convert the hydrophobic PLA electrospun membrane into a hydrophilic PLA membrane with controlled wettability, we mixed a blended solution of PLA and PEG-PPG-PEG in the pre-electrospinning stage. For electrospinning, the prepared PLA/PEG-PPG-PEG blend solutions were added separately to a 10 mL syringe with a 23-gauge needle, and the electrospinning machine was run at maximum capacity (30 kV, NF-500, MECC, Fukuoka, Japan) until the contents of the syringe were completely consumed. In this study, the applied voltage was 18 kV, the flow rate was 0.8 mL·h^−1^, the humidity was 10%, the temperature was ~24 °C, the distance between the needle tip and collector was 20 cm, and the drum collector was used with a rotational speed of 250 rotations per minute.

After electrospinning, the nanofiber membranes were removed from the collector, dried overnight in an electric oven at 45 °C, and then stored for characterization. The membranes were labeled according to their polymeric concentrations, as listed in Table 1.

### 2.4. Characterization of the Prepared Membranes

The morphology of the prepared electrospun membranes was studied using a field-emission scanning electron microscope (JSM-7600, JEOL, Tokyo, Japan). The membrane specimens were cut into small pieces and fixed onto a stub using carbon tape. The stubs were then coated (Auto Fine Coater, JFC-1600, JEOL, Tokyo, Japan) with platinum to increase the electrical conductivity of the sample during analysis. Subsequently, the coated samples were analyzed by FE-SEM under a high vacuum. The diameters of the NFs were calculated using Adobe Photoshop software. Here, we measured the diameters of 50 randomly chosen NFs. Photoshop was then used to measure the NF diameter based on the scale bar on the FE-SEM micrograph and convert it into a number.

A CA goniometer (OCA 15EC, Data Physics) was used to study the wettability of the nanofibrous membranes. A 4 ± 0.5 μL droplet of deionized water was placed on the membrane surface, and the angle between the liquid and surface was measured. At least three CA measurements were collected from different locations on each sample. The functional groups, amorphous or crystalline structures, and thermal behavior of the prepared PLA-based membranes were also identified. A Fourier-transform infrared spectrometer (VERTEX-70, Bruker, Billerica, MA, USA) was used to assess the functional groups of the membranes; the spectra were collected over the wavenumber range of 600–4000 cm^−1^ with 16 scans. The thermal behavior of the membranes was observed using a thermal gravimetric analyzer (Q600, TA Instruments, New Castle, DE, USA). TGA was performed at a heating rate of 10 °C·min^−1^ from 25 to 600 °C under an inert N_2_ atmosphere. The structures of the different PLA-based membranes were examined using an X-ray diffractometer (XRD-7000, Shimadzu, Kyoto, Japan). The XRD patterns were collected over the 2θ range of 2.0°–50° with continuous scanning at a rate of 2°·min^−1^. 

To measure the water stability of the nanofiber membranes, we cut each sample into squares, weighed ( $W_{i}$ ) them, and placed them on Petri dishes containing distilled water for 1, 7, 30, and 60 d at room temperature. At the indicated time interval, the nanofiber membranes were removed from the Petri dish and dried at room temperature for 24 h. The dry weight ( $W_{f}$ ) of the membranes was measured to determine their weight loss. Each sample was subjected to five wet–dry cycles to determine its reusability. The degree of water stability (%) was calculated using Equation (1) [29]:

(1) $$Stability\;\left(\%\right)=\left(\frac{W_{f}}{W_{i}}\right)\times 100.$$

The porosities of #S0, #S1, #S2, #S3, #S4, and #S5 were determined directly by calculating their pore ratio. The porosity percentage ( ε ) of the electrospun nanofiber membranes was calculated using Equation (2) [30]:

(2) $$\varepsilon \;\left(\%\right)=1-\frac{m}{\rho_{poly\;}\times A\times \delta }\times 100,$$

where m , $\rho_{poly\;}$ , A , and δ are the mass, density, surface area, and thickness of the membrane, respectively. To calculate the membrane surface areas, we cut the membranes into 3.5 × 2.5 cm^2^ pieces. Membrane thickness was measured using a thickness gauge (Ecotest Plus, Sheen Instruments, Cambridge, UK), and the average membrane thickness of each sample was calculated from at least 10 measurements taken at various locations on each sample. The density of the nanocomposite membranes was calculated by converting the PLA and PEG-PPG-PEG percentile contributions into decimal numbers (0–1) by dividing them by 100. Each decimal number was then multiplied by the densities of PLA and PEG-PPG-PEG individually, and all values were summed.

The WTRs of #S0, #S1, #S2, #S3, #S4, and #S5 were determined by measuring their weights before and after complete water absorption. We cut each membrane into squares and positioned them vertically such that the bottom side of the membrane was in direct contact with a methyl orange solution to visualize the time-dependent movement of the water. When the membrane had become completely wet, the WTR was evaluated by using Equation (3) [31]:

(3) $$WTR=\left(\frac{W_{W}-W_{D}}{A\times t}\right),$$

where $W_{w}$ and $W_{D}$ are the weights of the wet and dry membranes, respectively, A is the effective area of the membrane, and t is the time required for the membrane to become fully wet.

### 2.5. Fog Water-Collection System

**Figure 2:**
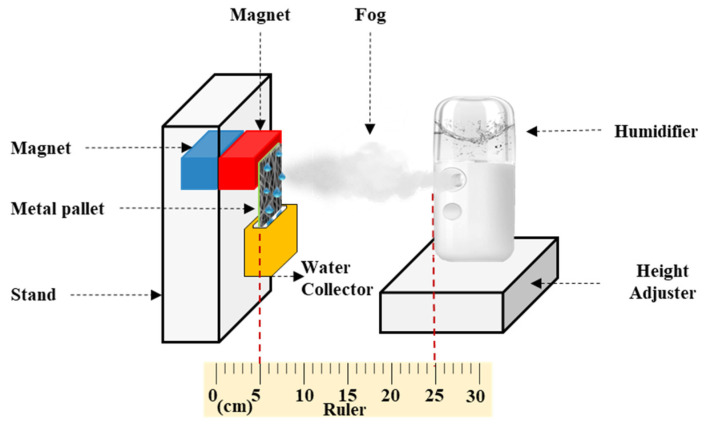
Schematic of the fog collection experiment.

Figure 2 shows a schematic of the configuration employed in the fog collection experiment. A humidifier (V-31, SIXTHGU, Shenzhen, China) with a flow rate of 230 m^3^·h^−1^ was fixed 20 cm from the surface of the membrane. Samples of #S0, #S1, #S2, #S3, #S4, and #S5 with an area of 30.25 cm^2^ (5.5 cm × 5.5 cm) were wrapped onto the iron plate. The iron plate was then connected to the magnets on the stand. The container was positioned below the membrane to collect the water produced from the fog. The fog was collected over a period of 1 h at intervals of 6 min. The relative humidity was kept at 15%, and the temperature was held at 25 °C.

## 3. Results and Discussion

### 3.1. Morphological Study

**Figure 3:**
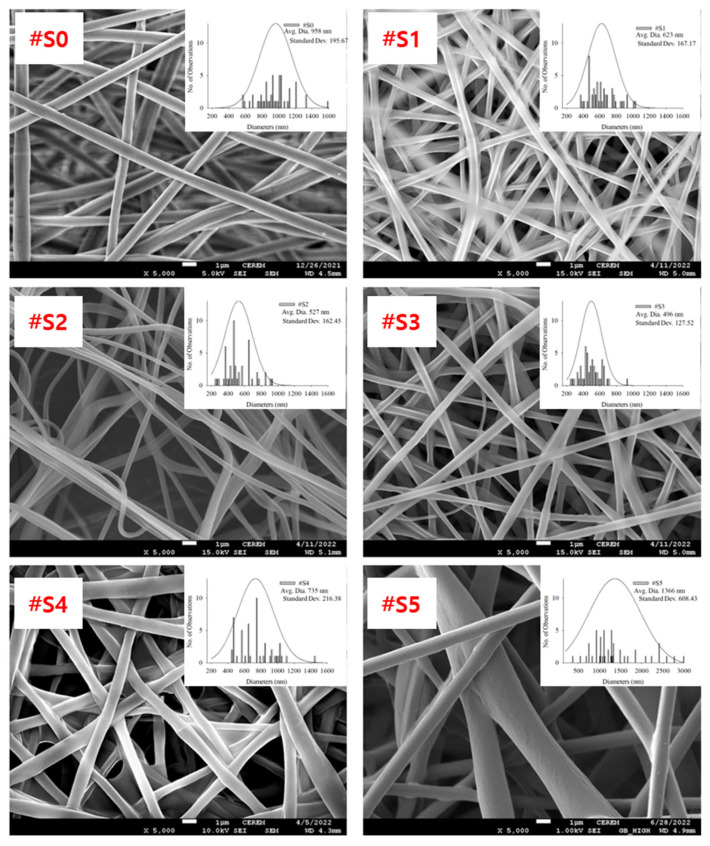
FE-SEM images, average diameters, and diameter distributions of (#S0), (#S1), (#S2), (#S3), (#S4), and (#S5).

FE-SEM images of the electrospun PLA and PLA/PEG-PPG-PEG blended nanofibers, their average diameters, and diameter distributions are shown in Figure 3.

The homopolymeric (#S0) nanofibers showed a uniform morphology. The thickness of the blended nanofibers showed notable changes as their PEG-PPG-PEG content increased. The nanofiber thickness can be altered by modifying the molecular weight or viscosity of the PEG-PPG-PEG solution. As the molecular weight and viscosity of the spinning solution increased, the diameter of the fibers tended to increase [32]. It was formed when two or three fibers were attached because the force required to separate the fibers in shapes of #S1 (the shape of churros) and #S2 (the cross-section of a dumbbell) is less than the force required to keep them together. #S3 showed the smallest average diameter (~496 nm, Figure 3) and a generally uniform shape.

### 3.2. Hydrophilicity Study

**Figure 4:**
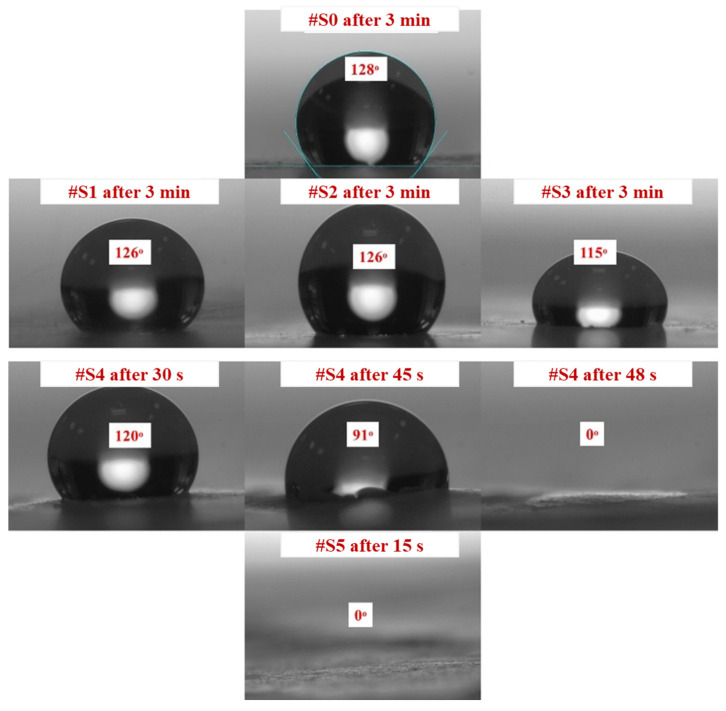
Contact angles of #S0; #S1, #S2, and #S3 after 3 min; #S4 after 30, 45, and 48 s; and #S5 after 15 s.

CA measurements confirmed that all samples had a certain degree of hydrophilicity. Figure 4 shows how the PEG-PPG-PEG block copolymer composition affects the CA of the different samples. The initial CA decreased as the PEG-PPG-PEG block copolymer content increased, which is consistent with the data obtained after CA monitoring for 3 min. High contact angles of 115° or more were observed in #S0, which is composed of pure PLA, and #S1, #S2, and #S3, which were composed of small amounts of the PEG-PPG-PEG block copolymer. The CA of #S4, at 120°, was fairly high in the first 30 s of monitoring but dropped to 0° as time progressed to 48 s. This phenomenon can be explained by the increase in surface hydrophilic groups owing to the increased concentration of the block copolymer [33]. In the presence of a large number of surface hydrophilic groups, water on the surface of the membrane can quickly spread along the nanofibers, penetrate the membrane pores, and wet the bottom surface of the membrane despite the poor water-sorption properties of PLA because of its hydrophobic nature [34,35]. Similarly, because of the presence of an excess amount of the PEG-PPG-PEG block copolymer, #S5 appeared to have high hydrophilicity.

### 3.3. Chemical Structure Analysis

**Figure 5:**
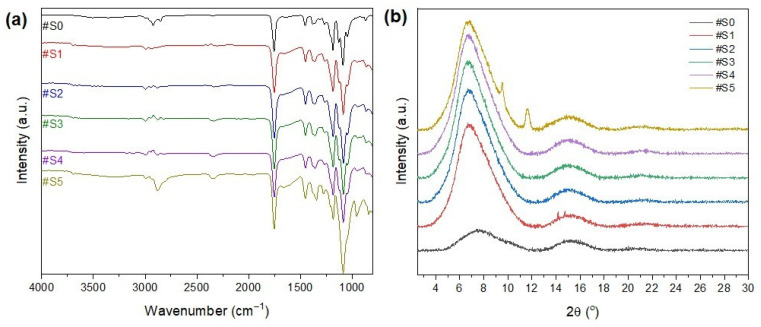
(**a**) FT-IR spectra and (**b**) XRD patterns of #S0, #1S, #2S, #3S, #S4, and #S5.

The FT-IR spectra of the nanofibrous membranes are shown in Figure 5a. The peak at 3200–3600 cm^−1^ could be attributed to -OH, which is the end group of both PLA and PEG-PPG-PEG. In general, the polymers are mainly composed of hydrophobic groups, and only the PEG terminals have hydrophilic groups. Thus, the hydrophilicity of the membranes increased with the amount of the copolymer added to the electrospinning solution. The peaks at 2950 and 2780 cm^−1^ could be attributed to C–H stretching vibrations, and their intensity increased as the PEG-PPG-PEG content increased. The peak at 1000–1300 cm^−1^ could be attributed to C–OC, and its intensity increased as the PEG-PPG-PEG content increased. The peak at 800–1000 cm^−1^ could be attributed to C–H bending vibrations. The peaks that are frequently found in PLA and PEG indicate the presence of other substances, thereby confirming the successful blending of PLA and PEG-PPG-PEG.

The XRD profiles of the electrospun nanofibers are shown in Figure 5b. The presence of two broad amorphous peaks at approximately 8° and 16.8° in the XRD patterns of the PLA/PEG-PPG-PEG-containing samples confirmed the amorphous microstructure of PLA [36]. The characteristic diffraction peaks of PEG were observed at 2θ = 19.2° and 23.2°, which could be assigned to the lattice planes of (115) and (016), respectively [37], and a typical halo at around 21° appeared in the XRD pattern of PPG owing to its amorphous nature [38]. The principal peaks of PPG (2θ = 19.2° and 23.2°) and PEG (2θ = 21°) did not appear in the XRD patterns of the PLA/PEG-PPG-PEG composite nanofiber membranes because of the low PEG/PPG ratio with respect to the matrix polymer PLA. The natural framework structure of PLA was significantly altered after the incorporation of PEG-PPG-PEG (#S1–#S5), and new peaks appeared at 2θ = 9° and 11°, corresponding to the (110) plane, at high concentrations of PEG-PPG-PEG (#S5). This finding confirms the successful dispersion of PEG-PPG-PEG into the PLA composite nanofibers. The characteristic peaks of PLA at around 8° and 16.8° were almost completely preserved in the XRD patterns of the blended nanofiber membranes, and only their intensity changed with increasing PEG-PPG-PEG content. A slight peak shift was also observed. The intensity of the diffraction peak increased with increasing PEG-PPG-PEG content in #S4, thereby confirming the increased crystallization of nanofibers in the PLA/PEG-PPG-PEG membranes. However, in the XRD pattern of #S5, the peak intensity decreased and the peak became wider, thus suggesting an increase in amorphous phases owing to the increase in PEG-PPG-PEG in the amorphous regions of the nanofiber membrane [39].

### 3.4. Thermal Properties

**Figure 6:**
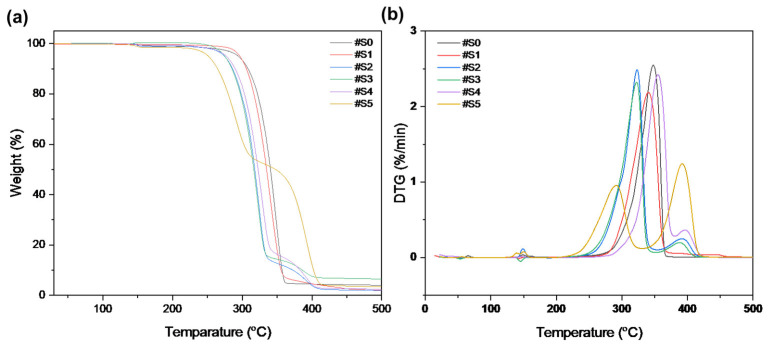
(**a**) TGA and (**b**) DTG curves of #S0, #1S, #2S, #3S, #S4, and #S5.

The thermal stability of the pure PLA and PLA/PEG-PPG-PEG blended nanofibers was examined using TGA. Figure 6 displays the weight loss and derivative weight loss (DTG) curves of the PLA and PLA/PEG-PPG-PEG nanofibers; these curves reveal details on the mechanism and degree of material degradation. The temperatures of the onset of thermal degradation (*T_onset_*) and maximum thermal degradation (*T_peak_*_1_ and *T_peak_*_2_) were assessed from these curves (Table 2). As shown in Figure 6a, the thermal degradation of #S0 and #S1 occurred at approximately the same temperature. The initial thermal degradation temperature of the PLA phase was lowered by the incorporation of PEG-PPG-PEG in #S2 and #S3, and decomposition occurred at approximately the same temperature. According to the DTG curves in Figure 6b, #S3–#S5 showed two decomposition peaks between 200 and 425 °C. The first decomposition peaks of #S2–#S4, at approximately 321–322 °C, and #S5, at 291.11 °C, represent the thermal decomposition of the PLA matrix, while the second decomposition peaks (388–391 °C) of #S2–#S5 indicate the thermal decomposition of PEG-PPG-PEG [40]. The decomposition peak of pure PLA shifted to a lower temperature in #S1–#S3, increased in #S4, and subsequently decreased in #S5. This fluctuation of the PLA decomposition peak may be due to the extent of lubrication of the PLA molecule, which depends on the PEG-PPG-PEG content. The high thermal decomposition peak of #S5 may be due to the plasticization of PLA by PEG-PPG-PEG, which reduces the crystallinity of the polymer at high concentrations [41]. The decomposition peak of PEG-PPG-PEG between 320 and 410 °C [42] slightly shifted toward higher values with increasing PEG-PPG-PEG content (#S1–#S5) in the PLA matrix. Moreover, according to the DTG curves, the #S1 is approximately 1 °C more stable with the thermal stability of the PLA nanofiber, indicating the improved compatibility of the composite components. These changes in the TGA/DTG curves of the samples confirmed that the necessary PLA/PEG-PPG-PEG (#S0–#S5) structures were obtained.

### 3.5. Water Stability

**Figure 7:**
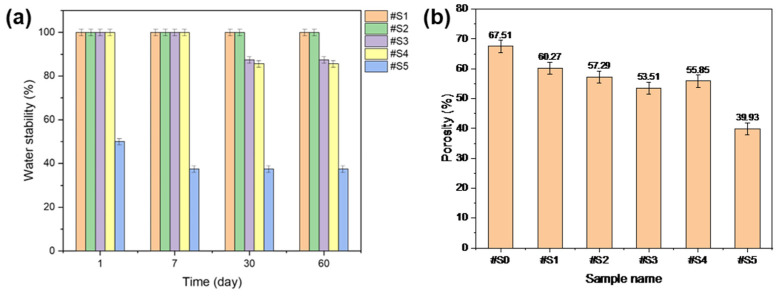
(**a**) Water stability and (**b**) porosity of the electrospun PLA and PLA/PEG-PPG-PEG nanofibers.

The water stability of the fabricated membranes was tested, and the results are shown in Figure 7a. #S1 and #S2 were highly stable in the presence of water. #S3 and #S4 showed sustained stability for up to 7 d; however, when the test was run for over 30 d, the stability of these samples dropped to ~80%. #S5 initially showed a stability of ~50%, but this value gradually decreased to ~40% over time.

### 3.6. Membrane Porosity

The porosity results of the prepared nanofibers are shown in Figure 7b. The porosity of the nanofibers generally decreased with increasing PEG-PPG-PEG content. Among the membranes, #S5 showed the lowest porosity, which could be assumed to be a result of its loose structure owing to its thick fibers. The lowest porosity, at approximately 55%, was observed in #S3. While the nanofibers produced in this sample were relatively dense, its porosity may be anticipated to be the lowest among the prepared membranes because its fiber diameter was the smallest.

### 3.7. Water Transport Rate

**Figure 8:**
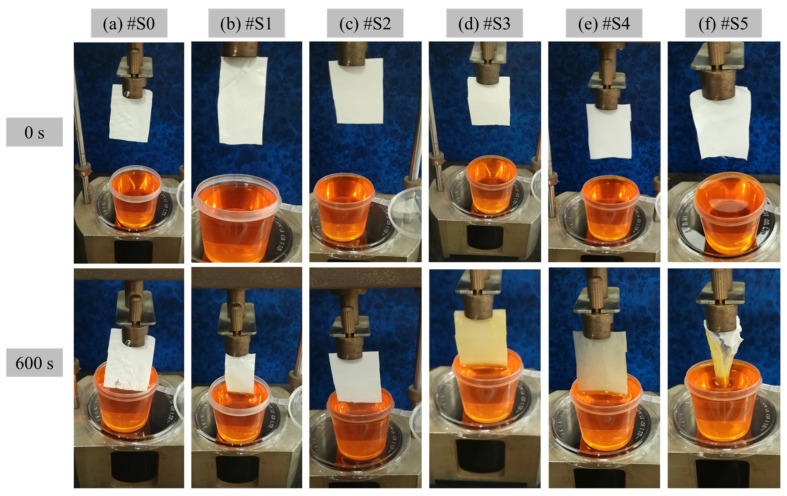
WTR of the electrospun (**a**) PLA (#S0) and (**b**–**f**) PLA/PEG-PPG-PEG-blended (#S1–#S5) nanofibers at 0 and 600 s.

The hydrophilicity of the prepared PLA and PLA/PEG-PPG-PEG membranes was verified in terms of WTR, and the experimental results are shown in Figure 8. The membranes were positioned such that they were in direct contact with the dye solution to observe the movement of the liquid and assess their hygroscopicity. Figure 8 clearly displays the amounts of water absorbed by the membranes from 0 to 600 s. Owing to the low hydrophilicity of #S0–#S2, the membranes did not begin to absorb water until 600 s had passed. By contrast, the colors of #S3 and #S4 changed visibly, and their WTR values were 3 and 8.1 mg·mm^−2^·h^−1^, respectively. After the membranes absorbed water, #S4 was more transparent than #S3. In the case of #S5, after 600 s, the membrane was damaged by the absorbed water; thus, its WTR could not be determined.

### 3.8. Water-Collection System

The water was collected from the captured fog as follows: (1) *Capture:* The collected fog was condensed on the collector surface. (2) *Collection:* The condensed water was coalesced into droplets. (3) *Transport:* The mass of the coalesced water droplets increased with their volume and induced droplets to travel to the end of the collector under the force of gravity [43].

**Figure 9:**
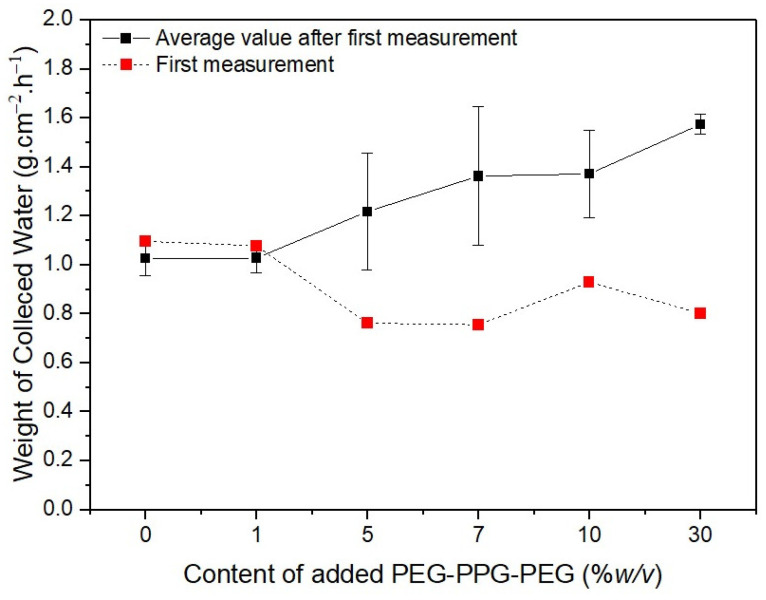
Weight of water collected over 6 min depending on the PEG-PPG-PEG content of the samples.

The red dotted lines in Figure 9 represent the initial weights of the water collected by the membranes. The water initially collected by #S0 and #S1 was remarkably similar, at approximately 1.1 g·cm^−2^·h^−1^. The volume of collected water marginally decreased as the PEG-PPG-PEG content increased, as observed in #S2 and #S3. The quantity of water on the surface of the nanofibers increases as their hydrophilicity increases. In comparison with that on #S0 and #S1, the amount of water on the nanofiber surfaces of #S2 and #S3 increased because the distance between nanofibers in these membranes was larger owing to their higher porosity. Thus, the amount of water collected from #S2 and #S3 was generally less than that collected from #S0 and #S1. As more water vapor condenses, the water droplets fill the spaces between the nanofibers and fall to the ground because of gravity. Thus, the actual amount of water collected is equal to the amount of water collected on the membranes minus the portion absorbed by the nanofibers. According to the water CA images shown in Figure 4, absorption begins at 48 s. Thus, the amount of water collected from #S4 is slightly higher than that collected from #S2 and #S3. When the PEG-PPG-PEG content was increased, as in #S5, the hydrophilicity of the nanofibers significantly increased, and the amount of water vapor condensed on the nanofiber surface increased correspondingly. However, because the nanofibers absorbed a large amount of water, the amount of water collected from #S5 decreased slightly compared with that collected from #S4.

The black solid line in Figure 9 shows the results of five additional measurements taken after the first collection was completed. The amount of water collected in the second cycle of collection differed from that in the first cycle of collection because the second test was performed just before the membranes were dried. In this case, the quantity of collected water tended to increase as the PEG-PPG-PEG content increased. The difference in collected amounts was obvious in samples #S2, #S3, and #S4 because of the rate at which the hydrophobic portion of the membranes was cleared of the water vapor that had been absorbed by the fibers in their wet state. The rate of volume increase of the coalesced water droplets increased with the amount of water vapor still present in the pores, leading to a higher collection rate. Because the rate of absorption into the fibers was faster than that in #S2 or #S3, the amount of water absorbed progressively increased as the setting began. As a result, the difference in water collected decreased in #S4. The difference in fog collection quantity was the smallest for #S5. Because the pores of #S5 are smaller than those of the other samples, we believe that repeating the experiment will only have a small impact on the amount of water collected from this sample.

## 4. Conclusions

PLA nanofibrous membranes were modified using PEG-PPG-PEG tri-block copolymers for the first time via the pre-electrospinning technique. The PEG-PPG-PEG content was adjusted to regulate the hydrophilicity of the PLA-based nanofibrous membranes. The thickness of the prepared nanofibers tended to change as the PEG-PPG-PEG content increased, and we selected samples #S3 and #S4 as the most appropriate nanofibrous membranes for our purpose. The CAs of the membranes decreased as their PEG-PPG-PEG content increased, and the CA of #S4 decreased to 0° after 48 s. FT-IR and XRD analyses confirmed that PEG-PPG-PEG was well contained in the membranes by blending. The water stability of #S3 and #S4 was sustained near 99% for up to 7 d and remained greater than 80% even after over 60 d. The porosity of the nanofibers generally decreased with increasing PEG-PPG-PEG content. The colors of #S3 and #S4 changed visibly, and their WTR values were 3 and 8.1 mg·mm^−2^·h^−1^, respectively. As the PEG-PPG-PEG content of the membranes increased, the weight of the collected water increased by over 40% compared with that captured by the pure PLA membrane. In addition to water harvesting, the prepared blends could improve and broaden the use of PLA-based membranes in applications such as tissue engineering, drug delivery, scaffolds, and pharmaceuticals.

## Figures and Tables

**Table 1:** Labels and compositions of the PLA and PLA/PEG-PPG-PEG solutions.

Sample Name	Concentration of PLA (%, *w*/*v*)	Concentration of PEG-PPG-PEG (%, *w*/*v*)	Spin.-Blend Solution Composition
#S0	12	0	4:1 ratio of the PLA and PEG-PPG-PEG solutions
#S1		1	
#S2		5	
#S3		7	
#S4		10	
#S5		30	

**Table 2:** TGA results of #S0, #1S, #2S, #3S, #S4, and #S5.

Sample Name	*T_onset_* (°C)	*T_peak_*_1_ (°C)	*T_peak_*_2_ (°C)
#S0	260.93	347.49	-
#S1	260.92	341.20	-
#S2	237.49	322.9	391.44
#S3	234.28	322.9	388.07
#S4	266.07	321.13	391.44
#S5	222.56	291.11	391.44

## Data Availability

Not applicable.
